# Higher risk of revision for partial knee replacements in low absolute volume hospitals: data from 18,134 partial knee replacements in the Dutch Arthroplasty Register

**DOI:** 10.1080/17453674.2020.1752017

**Published:** 2020-04-14

**Authors:** Iris van Oost, Koen L M Koenraadt, Liza N van Steenbergen, Stefan B T Bolder, Rutger C I van Geenen

**Affiliations:** a Foundation for Orthopedic Research, Care & Education, Amphia Hospital, Breda;; b Dutch Arthroplasty Register (LROI), ‘s-Hertogenbosch;; c Department of Orthopedic Surgery, Amphia Hospital, Breda, the Netherlands

## Abstract

Background and purpose — Partial knee replacement (PKR) survival rates vary a great deal among registries and cohort studies. These discrepancies can largely be attributed to inappropriate indications of the PKR and low thresholds for revision, but also to the PKR volume. This study used Dutch Arthroplasty Register data to analyze whether absolute PKR or proportional PKR hospital volume is associated with the risk of revision.

Patients and methods — 18,134 PKRs were identified in the Dutch Arthroplasty Register from 2007 to 2016. For each year, hospitals were divided into 4 groups based on the quartiles for the absolute volume (< 22, 22–36, 36–58 and > 58 PKRs per year) and the proportional volume (< 8.5, 8.6–14.2, 14.3–25.8 and > 25.8% PKRs). Kaplan–Meier survival analysis was performed to determine survival rates. A multivariable Cox regression adjusted for age category, sex, ASA score, year of surgery, diagnosis, unicondylar side, and type of hospital was used to estimate hazard ratios (HR) for revision.

Results and interpretation — Proportional PKR volume did not, but absolute PKR volume did influence the risk of revision. The adjusted HR for hospitals with an absolute volume of 22–36 PKRs per year was 1.04 (95% CI 0.91–1.20), 0.96 (CI 0.83–1.10) for the hospitals with 36–58 PKRs, and 0.74 (CI 0.62–0.89) for hospitals with more than 58 PKRs compared with hospitals that had fewer than 22 PKRs per year. So, patients treated with a PKR in a high absolute volume hospital have a lower risk of revision compared with those treated in a low absolute volume hospital.

One of the advantages of partial knee replacement (PKR) is that the native mechanics of the knee are largely preserved, whereas in total knee replacement (TKR) the anterior cruciate ligament is sacrificed and the mechanics change substantially (Laurencin et al. 1991). This might contribute to better a postoperative clinical outcome following PKR compared with TKR (Liddle et al. 2015). Also, a lower risk of complications has been reported for PKR (Liddle et al. 2014a, Beard et al. 2019). Furthermore, a recent randomized trial demonstrated similar Oxford Knee Scores, a higher perceived knee improvement, higher willingness to undergo the operation again, and better cost-effectiveness after PKR compared with TKR at 5 years’ follow-up (Beard et al. 2019). Nevertheless, since its introduction, the PKR has been a topic of debate due to the diversity in reported long-term survival rates.

Multiple studies have reported survival rates over 94% at 10 years (Svärd and Price 2001, Pandit et al. 2011, Lisowski et al. 2011, Burnett et al. 2014). However, registries and low-volume PKR centers showed significantly lower survival rates at 5 and 10 years (Baker et al. 2012, Schroer et al. 2013, Badawy et al. 2017). The discrepancies in survival rates can largely be attributed to low absolute and proportional PKR volume (Liddle et al. 2015, Badawy et al. 2017). Badawy et al. showed that in the Nordic countries the most common annual absolute hospital volume was 1–3 PKRs per year, which could have resulted in the reported low survival rates.

The use of PKR is generally accepted in the Netherlands. However, large variation in knee replacement volume exists between hospitals. Data from high-volume and low-volume hospitals are available in the Dutch Arthroplasty Register (LROI)(Van Steenbergen et al. 2015). The LROI database contains data concerning orthopedic joint implants in the Netherlands since 2007. This study investigates whether absolute PKR hospital volume and proportional PKR hospital volume are associated with higher risk of revision using population-based national register data.

## Patients and methods

### Study population

The Dutch Arthroplasty Register database contains data on orthopedic joint implants in the Netherlands since 2007. The completeness of the LROI database is more than 95% for primary knee arthroplasty with a coverage of all hospitals in the Netherlands (Van Steenbergen et al. 2015). Data were extracted from the Dutch Arthroplasty Register database for all primary TKR and PKR procedures between 2007 and 2016, including bilateral procedures. 18,134 PKRs and 190,204 TKRs were registered in this period. For each patient, data were gathered regarding surgical characteristics (e.g., implantation of TKR or PKR, year of operation, type of hospital (university medical center, general hospital, independent treatment center), and anonymized hospital) and patient characteristics (e.g., age, sex, ASA score, diagnosis, and previous surgery on the affected knee).

### Data analyses

Follow-up was defined as time between primary procedure until revision, death, or end date of follow-up (January 1, 2017). Revision was defined as every change (placement, replacement, or removal) of one or more components of the knee prosthesis. Per year, the absolute and proportional PKR volume was determined for each hospital. These volumes were determined by calculating the PKR volume per year (absolute PKR volume) and TKR volume of each hospital. Subsequently, the percentage of PKRs was calculated for the total number of knee arthroplasties (PKR/(TKR + PKR)*100; proportional PKR volume). The volumes across all years and hospitals were divided into 4 groups based on the quartiles. With respect to the absolute PKR volume, the PKRs were classified into < 22, 22–36, 36–58, or > 58 PKRs per year. For the proportional PKR volume, each PKR was classified into < 8.5, 8.6–14.2, 14.3–25.8, or > 25.8% PKRs per year. The median follow-up of PKRs was 4.0 years (IQR 2.2–6.9).

### Statistics

Kaplan–Meier survival analyses were performed to determine survival rates at 4-year and 8-year follow-up. These survival analyses were performed for both absolute and proportional hospital volumes, with revision for any reason as endpoint. The follow-up started on the day of the primary PKR procedure and ended on the day of first revision, death, or the end of the follow-up period. Differences in patient and procedure characteristics of both absolute and proportional hospital volumes were assessed by Pearson’s chi-square test.

Univariable and multivariable Cox regression models were conducted. Spearman’s rho was calculated to estimate the correlation between the absolute and proportional volume of the hospitals. In case of a strong correlation (rho > 0.6) the absolute and proportional hospital volumes are analyzed in separate univariate and multivariable Cox regressions.

Cox regression analyses were used to estimate hazard ratios (HRs) with a 95% confidence interval (CI) to investigate the association between absolute and proportional volume and the survival of the PKRs. In the multivariable Cox regression model the HRs were adjusted for age category, sex, ASA score, year of surgery, diagnosis, unicondylar side, and type of hospital. These factors can independently influence the risk of revision following PKR. Residual confounding was investigated with a sensitivity analysis. As a sensitivity analysis, Cox models were performed for every individual variable, for all patient-level variables together and also separately for the hospital level variable, type of hospital. HRs were presented relative to the lowest absolute and proportional volume group. For all covariates added to the model, the proportional hazards assumption was checked by inspecting log-minus-log curved and met. P-values < 0.05 were considered significant. For the 95% confidence intervals (CI) we assumed that the number of observed cases followed a Poisson distribution. The statistical package SPSS (version 25, IBM Corp, Armonk, NY, USA) was used for all statistical analyses.

### Ethics, funding, and potential conflicts of interest

As the study was based on registry data, ethical approval was not needed. This study received no funding and the authors declare no conflicts of interest regarding this study.

## Results

98 hospitals performed 190,204 TKRs and 18,134 PKRs between 2007 and 2016 in the Netherlands. The patient and procedure characteristics for the absolute as well as the proportional volume groups are shown in Tables 1 and 2. The median number of PKRs performed per hospital was 36 per year (IQR = 22–58). With regard to the proportional hospital volume, the median percentage of knee arthroplasties performed with a PKR was 14.2%. Spearman’s correlation test showed a strong correlation between the absolute and proportional hospital volumes (rho = 0.69).

**Table 2.** Patient and procedure characteristics of the 4 proportional hospital volume groups from 2007 to 2016. Values are number (%) unless otherwise specified

Factor	Proportional hospital volume groups				p-values
	< 8.5%	8.5–14.2%	14.2–25.8%	> 25.8%	
	n = 4,535	n = 4,576	n = 4,563	n = 4,460	
Age, mean (SD)	61 (9)	63 (9)	63 (9)	63 (9)	< 0.001
Age group					< 0.001
< 55	1,027 (22.7)	831 (18.2)	772 (16.9)	811 (18.2)	
55–64	2,025 (44.7)	1,854 (40.6)	1,870 (41.0)	1,702 (38.2)	
65–74	1,202 (26.5)	1,440 (31.5)	1,458 (32.0)	1,439 (32.3)	
≥ 75	276 (6.1)	442 (9.7)	462 (10.1)	504 (11.3)	
Men	(42)	(39)	(42)	(41)	< 0.01
ASA grade					< 0.001
I	1,576 (34.8)	1,428 (31.2)	1,297 (28.4)	1,670 (37.4)	
II	2,489 (54.9)	2,590 (56.6)	2,573 (56.4)	2,420 (54.3)	
III–IV	230 (5.1)	302 (6.6)	360 (7.9)	275 (6.2)	
Diagnosis					< 0.05
Osteoarthritis	4,423 (97.5)	4,483 (98.0)	4,487 (98.3)	4,396 (98.6)	
Other	52 (1.1)	39 (0.9)	28 (0.6)	53 (1.2)	
Unicondylar side					< 0.001
Medial	3,935 (86.8)	4,089 (89.4)	4,087 (89.6)	4,020 (90.1)	
Lateral	66 (1.5)	48 (1.0)	115 (2.5)	262 (5.9)	
Year of surgery					< 0.001
2007–2010	1,421 (31.3)	1,542 (33.7)	1,224 (26.8)	1,037 (23.2)	
2011–2013	1,531 (33.3)	1,111 (24.3)	1,284 (28.1)	1,041 (23.3)	
2014–2016	1,601 (35.3)	1,923 (42.0)	2,055 (45.1)	2,382 (53.4)	

**Table 1.** Patient and procedure characteristics of 18,134 PKRs according to 4 absolute hospital volume groups from 2007 to 2016. Values are number (%) unless otherwise specified

	Absolute hospital volume groups				
	< 22	22–36	36–58	> 58	
Factor	n = 4,734	n = 4,559	n = 4,448	n = 4,393	p-value
Age, mean (SD)	61 (9)	62 (9)	63 (9)	64 (9)	< 0.001
Age group					< 0.001
< 55	1,028 (21.8)	876 (19.2)	781 (17.6)	756 (17.2)	
55–64	2,126 (45.0)	1,925 (42.3)	1,841 (41.4)	1,559 (35.5)	
65–74	1,270 (26.9)	1,348 (26.9)	1,389 (31.2)	1,532 (34.9)	
≥ 75	302 (6.4)	407 (8.9)	434 (9.8)	541 (12.3)	
Men	(41)	(42)	(41)	(41)	> 0.05
ASA grade					< 0.001
I	1,722 (36.4)	1,551 (34.0)	1,537 (34.6)	1,161 (26.4)	
II	2,436 (51.5)	2,430 (53.3)	2,532 (56.9)	2,674 (60.9)	
III–IV	256 (5.4)	261 (5.7)	231 (5.2)	419 (9.5)	
Diagnosis					< 0.001
Osteoarthritis	4,566 (96.5)	4,423 (97.0)	4,353 (97.9)	4,314 (98.2)	
Other	93 (2.0)	79 (1.7)	63 (1.4)	68 (1.5)	
Unicondylar side					< 0.001
Medial	4,061 (85.8)	4,077 (89.4)	4,038 (90.8)	3,955 (90.0)	
Lateral	80 (1.7)	111 (2.4)	124 (2.8)	176 (4.0)	
Year of surgery					< 0.001
2007–2010	1,679 (35.4)	1,746 (38.4)	1,352 (30.4)	447 (10.2)	
2011–2013	1,586 (33.5)	1,155 (25.3)	1,340 (29.8)	868 (19.8)	
2014–2016	1,469 (31.0)	1,658 (36.3)	1,756 (39.5)	3,078 (70.1)	

**Figure 1.**
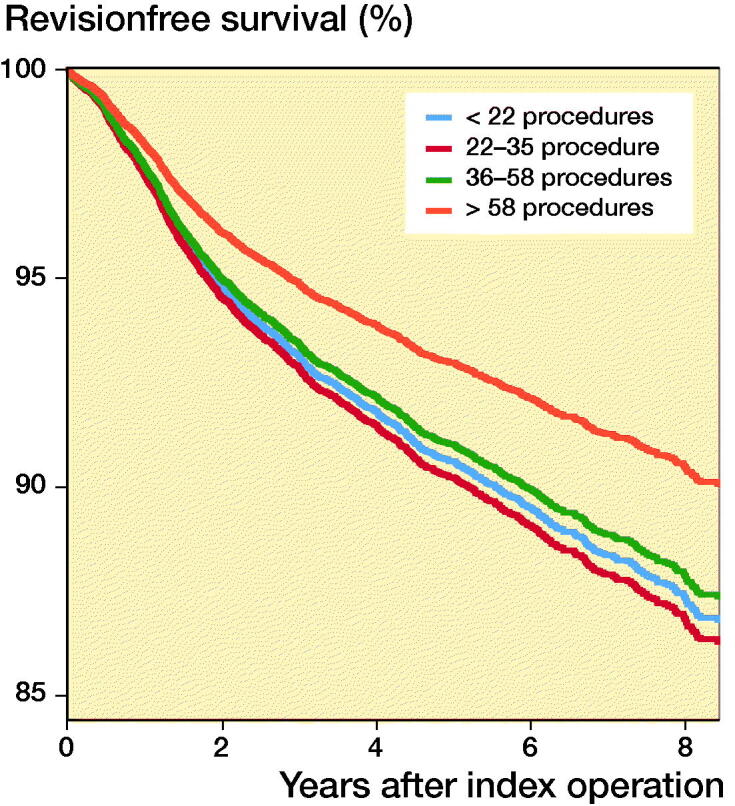
Cox regression survival curve for the absolute hospital volume adjusted for age category, sex, ASA score, year of surgery, diagnosis, unicondylar side, and type of hospital.

With regard to absolute volume, the 4-year survival was 90.9% for the lowest volume group (< 22 PKRs per year), 90.7% for the 22–36 PKRs per year group, 92.4% for the 36–58 PKRs per year group, and 93.5% for the highest absolute PKR volume hospitals (> 58 PKRs per year) (Table 3). The Kaplan–Meier estimated survival at 8 years’ follow-up had dropped to 86.7% for the lowest volume group (< 22 PKRs per year), 85.9% for the 22–36 PKRs per year group, 87.7% for the 36–58 PKRs per year group, and 89.3% for the highest absolute PKR volume hospitals (> 58 PKRs per year) (Table 3). The univariate Cox regression showed a statistically significant difference between the highest absolute volume group (> 58 PKRs per year) and the lowest absolute volume group (< 22 PKRs per year) with an HR of 0.74 (CI 0.63–0.86). Also, in the multivariable Cox regression model, the absolute hospital volume influenced the risk of revision (Table 4). The highest absolute volume group (> 58 PKRs per year) had a lower risk of revision compared with the lowest volume group (< 22 PKRs per year) with an adjusted HR of 0.74 (CI 0.62–0.89) (Figure 1 and Table 4). Other factors that influenced the risk of revision in the multivariable Cox regression for the absolute hospital volume groups were ASA classification, unicondylar side, and age (Table 4). ASA classification III–IV showed inferior results with an adjusted HR of 1.37 (CI 1.09–1.73) compared with ASA classification I. All age groups had a statistically significant reduced risk of revision compared with the lowest age group. The lateral PKRs showed a higher risk of revision compared with the medial PKRs with an HR of 1.32 (CI 1.02–1.71).

**Table 4.** Multivariable Cox regression results of the absolute hospital volume groups adjusted for age category, sex, ASA, year of surgery, diagnosis, unicondylar side, and type of hospital

Factor	HR (95%CI)	p-value
Absolute hospital volume		
< 22	1.0 (ref)	
22–36	1.04 (0.91–1.20)	0.6
36–58	0.96 (0.83–1.10)	0.5
> 58	0.74 (0.62–0.89)	< 0.005
Age		
< 55	1.0 (ref)	
55–64	0.68 (0.60–0.77)	< 0.001
65–74	0.53 (0.46–0.61)	< 0.001
≥ 75	0.39 (0.30–0.50)	< 0.001
Sex		
Male	1.0 (ref)	
Female	0.97 (0.87–1.08)	0.6
ASA		
I	1.0 (ref)	
II	1.10 (0.98–1.23)	0.1
III–IV	1.37 (1.09–1.73)	0.01
Year of surgery		
2007–2010	1.0 (ref)	
2011–2013	0.97 (0.88–1.14)	0.8
2014–2016	0.93 (0.80–1.08)	0.3
Diagnosis		
Osteoarthritis	1.0 (ref)	0.7
Other	1.10 (0.69–1.75)	
Unicondylar side		
Medial	1.0 (ref)	
Lateral	1.32 (1.02–1.71)	0.04
Unknown	1.36 (0.88–2.10)	0.2
Type of hospital		
General hospital	1.0 (ref)	
University center	0.87 (0.60–1.27)	0.5
Independent center	1.10 (0.95–1.28)	0.2

**Table 3.** Results from the 4- and 8-year KM survival analysis on the absolute hospital volume

Absolute hospital volume	Revisions n (%)	Deaths n (%)	K–M-4 year survival (95% CI)	K–M 8-year survival (95% CI)
< 22	507 (10.7)	150 (3.2)	90.9 (90.1–91.7)	86.7 (85.5–87.9)
22–36	482 (10.6)	144 (3.2)	90.7 (89.7–91.7)	85.9 (84.5–87.3)
36–58	390 (8.8)	169 (3.8)	92.4 (91.6–93.2)	87.7 (86.3–89.1)
> 58	252 (5.7)	97 (2.2)	93.5 (92.5–94.5)	89.3 (87.5–91.1)

**Figure 2.**
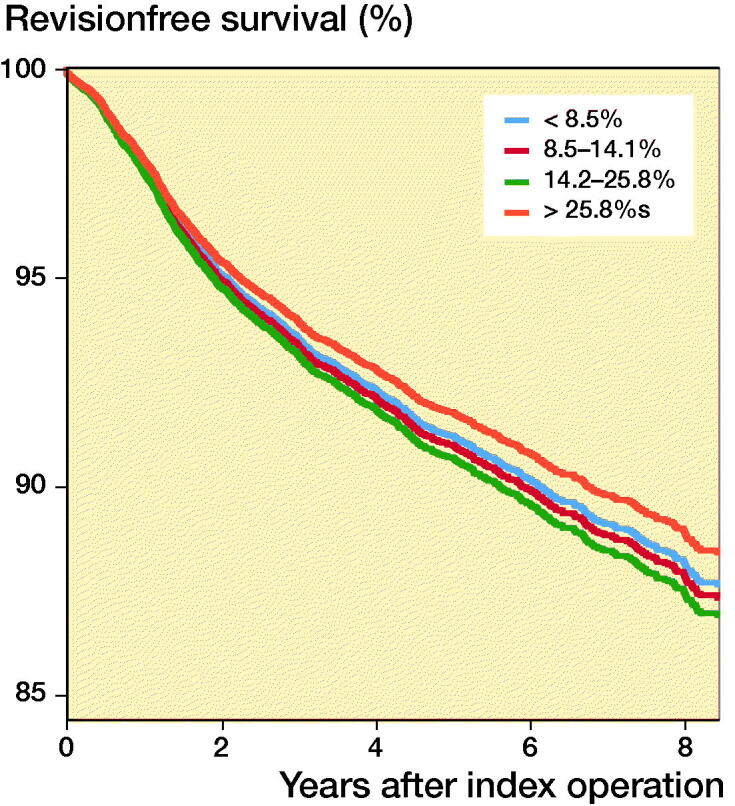
Cox regression survival curve for the proportional hospital volume adjusted for age category, sex, ASA score, year of surgery, diagnosis, unicondylar side, and type of hospital.

With regard to proportional PKR volume, the Kaplan–Meier 4-year survival was 91.3% for the lowest volume group (< 8.5% PKRs per year), 91.8% for the 8.5–14.2% PKRs per year group, 91.4% for the 14.2–25.8% PKRs per year group, and 92.7% for the highest proportional PKR volume group (> 25.8% PKRs per year). The Kaplan–Meier estimated survival at 8 years’ follow-up had dropped to 86.6% for the lowest proportional volume group (< 8.5% PKRs per year), 87.2% for the 8.5–14.2% PKRs per year group, 87.4% for the 14.2–25.8% PKRs per year group, and 87.8% for the highest proportional PKR volume hospitals (> 25.8% PKRs per year) (Figure 2 and Table 5). The univariate Cox regression revealed that the proportional hospital volume groups did not influence the risk of revision. Also in the multivariable Cox regression model the proportional hospital volume groups did not influence the risk of revision. Other factors that influenced the risk of revision in the multivariable Cox regression for the proportional hospital volume groups were ASA classification, age, and year of surgery (Table 6).

**Table 5.** Results from the 4- and 8-year KM survival analysis on the proportional hospital volume

Proportional hospital volume (%)	Revisions n (%)	Deaths n (%)	K–M-4 year survival (95% CI)	K–M 8-year survival (95% CI)
< 8.5	457 (10.1)	138 (3.0)	91.3 (90.5–92.1)	86.6 (85.2–88.0)
8.5–14.2	420 (9.2)	144 (3.1)	91.8 (91.0–92.6)	87.2 (85.8–88.6)
14.2–25.8	406 (8.9)	144 (3.2)	91.4 (90.4–92.4)	87.4 (86.0–88.8)
> 25.8	348 (7.8)	134 (3.0)	92.7 (91.9–93.5)	87.8 (86.4–89.2)

**Table 6.** Multivariable Cox regression results of the proportional hospital volume groups adjusted for age category, sex, ASA, year of surgery, diagnosis, unicondylar side, and type of hospital

Factor	HR (95%CI)	p-value
Proportional hospital volume		
< 8.5%	1.0 (ref)	
8.5–14.2%	1.03 (0.89–1.19)	0.7
14.2–25.8%	1.06 (0.92–1.24)	0.4
> 25.8%	0.93 (0.79–1.11)	0.4
Age		
< 55	1.0 (ref)	
55–64	0.68 (0.60–0.77)	< 0.001
65–74	0.52 (0.45–0.61)	< 0.001
≥ 75	0.38 (0.30–0.49)	< 0.001
Sex		
Male	1.0 (ref)	
Female	0.97 (0.87–1.08)	0.5
ASA		
I	1.0 (ref)	
II	1.08 (0.96–1.21)	0.2
III–IV	1.32 (1.04–1.66)	< 0.05
Year of surgery		
2007–2010	1.0 (ref)	
2011–2013	0.97 (0.85–1.11)	0.7
2014–2016	0.86 (0.75–1.0)	< 0.05
Diagnosis		
Osteoarthritis	1.0 (ref)	
Other	1.12 (0.70–1.78)	0.6
Unicondylar side		
Medial	1.0 (ref)	
Lateral	1.31 (1.01–1.70)	< 0.05
Unknown	1.36 (0.88–2.09)	0.2
Type of hospital		
General hospital	1.0 (ref)	
University center	0.93 (0.64–1.35)	0.7
Independent center	1.05 (0.89–1.23)	0.6

## Discussion

In this Dutch arthroplasty register study 98 hospitals performed more than 190,000 TKRs and over 18,000 PKRs with a median follow-up of around 4.0 years for the PKRs. The main finding was that hospitals performing more than 58 PKR procedures per year demonstrated a lower risk of revision compared with hospitals that had less than 22 PKR procedures. In contrast, proportional PKR volume was not associated with the risk of revision.

The association between absolute hospital PKR volume and survival found in our study is in line with findings of previous studies. However, these studies found annual hospital volumes of less than 12, 13, and 40 to be associated with a higher risk of revision, compared with 58 in our study (Baker et al. 2013; Badawy et al. 2014, 2017). The Cox regression analysis of Badawy et al. showed much lower HRs for the higher volume groups than our study, which indicates a stronger effect of hospital volume on risk of revision in their study (Badawy et al. 2014). In particular, for the group performing over 41 procedures annually the HR was 0.59, whereas in our study a HR of 0.74 was found for the group performing more than 58 procedures annually. This HR was also our only statistically significant HR, whereas in both studies of Badawy et al. all the volume groups had a significant lower HR compared with the lowest volume group (Badawy et al. 2014, 2017). This discrepancy might be the result of the lower survival rates observed in the Nordic studies, indicating that the low-volume groups in our study already perform quite well. In addition, in general the PKR volumes were much lower in the studies of Badawy et al., resulting in a lowest volume group with much lower PKR volumes compared with our study. Hence, although the effect was smaller compared with data from other registries, possibly as a result of more PKR use in the Netherlands and higher survival rates in general, higher absolute PKR volume still results in a lower risk of revision.

With regard to the proportional PKR volume, our study revealed no association with revision rate. The effect of proportional PKR volume on the risk of revision has been evaluated in 1 previous study (Liddle et al. 2015), which did find an association between proportional surgeon volume and the survival of PKRs: the lowest relative risk of revision in PKRs when 40–60% of the knee arthroplasties were performed with a PKR. The difference between our study and the study of Liddle et al. (2015) might be explained by the fact that they investigated the proportional volume on the level of the surgeon while this study focused on the proportional hospital volume. Unfortunately, we were not able to extract the data at the level of the surgeon so whether the proportional surgeon volume actually influences the survival based on the data in our study remains unknown. Assuming that a similar proportional surgeon volume effect is present in the data of our study, the reason the same effect was not observed for proportional hospital volume might be explained by in-hospital referral of patients eligible for PKR to the more specialized PKR knee surgeons. As a result, hospitals with 1 surgeon performing all PKRs can be assigned to the same proportional hospital volume group as hospitals with every surgeon performing a couple of PKRs. This while the situation in the former hospital is expected to be superior as a result of the positive association between absolute surgeon PKR volume and survival (Baker et al. 2013, Liddle et al. 2016). Hence, the possible positive effect of higher proportional surgeon volume is not reflected in higher proportional hospital volume. The assumption of the presence of a similar proportional surgeon volume effect compared with Liddle et al. in our study might, however, be doubted, especially because the absolute numbers of PKRs in their study per surgeon is very low with on average 2.8 PKRs per knee surgeon (Liddle et al. 2015). If we compare this with the situation of the country of our study, the average number of PKRs is already 3.6 when the number of PKRs is divided by the number of all practicing orthopedic surgeons. This includes also upper extremity surgeons. These absolute numbers directly influence the proportional surgeon volumes and therefore we expect the influence of proportional surgeon volume on prosthesis survival to be less obvious in the Netherlands compared with the UK. However, we were not able to clarify this. Therefore, future studies are needed to see if it is only the proportional surgeon or also the proportional hospital volume that influences the risk of revision and these studies should take the internal hospital referral policy into account. In addition to the finding that absolute hospital volume is related to the risk of revision, our study also revealed that younger patients have a higher risk of revision compared with older patients. Other studies have also shown a higher risk of revision in younger patients after PKR with similar findings in primary hip and total knee arthroplasty (Kuipers et al. 2010, Badawy et al. 2017, Bayliss et al. 2017). Data from other registers (AOANJRR 2018, NZJR 2018, SKAR 2018) support these findings. As shown in the study of Bayliss et al., the majority of revisions occur after a long period of follow-up, suggesting that the only reason for an increased risk of revision in younger patients results from the simple reason of longer follow-up in this group. Another reason, possibly more of influence in PKR than total knee and hip replacement, might be that PKRs are performed for the wrong indication in the younger patients. This hypothesis is supported by the fact that in our study the age was also significantly lower in the lower hospital volume groups and the fact that a study of the designer group did not revealed an effect of age on the risk of revision (Kennedy et al. 2018). Hence, both the longer period young patients depend on their prosthesis but also the possibility of low age as an indication for PKR might result in a higher risk of revision in younger patients.

Data gathering and data completeness are necessary for reliable and meaningful knee arthroplasty research. The completeness of the LROI for knee arthroplasty is more than 95% with a 100% coverage of hospitals (Van Steenbergen et al. 2015). However, a limitation of the LROI is the lack of historical information on individual surgeon volume. This lack of information on surgeon volume made it impossible to investigate the proportional surgeon volume and absolute surgeon volume. This is an issue mainly for the proportional volume question. However, the proportional hospital volume (as used in our study) may have advantages, because the over- or underestimation as a result of in-hospital referrals of proportional surgeon volume (used by Liddle et al. 2015) does not play a role and the hospital volume addresses the entire patient population of a hospital and reflects how a hospital has incorporated PKR. Nevertheless, to get a complete overview of the effect of proportional volume on survival we suggest evaluating both surgeon and hospital volume for the evaluation of internal hospital referral policy (Liddle et al. 2015). Another limitation is that residual confounding may persist in our data despite the performed adjustments. We could only adjust for factors present in the LROI database and therefore residual confounding may persist due to unmeasured and also imperfectly measured variables. An example of an unmeasured variable is the operating surgeon grade, which influences the implant survival as found by Liddle et al. (2014b). After the sensitivity analysis we concluded that the multivariable Cox regression adjusted for age category, sex, ASA score, year of surgery, diagnosis, unicondylar side, and type of hospital was the best fitted model with the least residual confounding.

Based on the knowledge available now regarding absolute and proportional hospital and surgeon PKR volume, we can state that the longevity of the prosthesis is positively associated with volume. Most studies focused on absolute volume (surgeon and/or hospital), resulting in several different cut-off levels ranging from 11–58 PKR per year for hospital volume and 10–13 PKR per year for surgeon volume with a clear tendency of even better survival rates with increasing volumes. With regard to the proportional volume this has only been proven on the level of the surgeon, with surgeons performing 40–60% of their knee arthroplasties with a PKR showing the best results. This might actually be a derivative of performing PKR for the proper indication, since Willis-Owen et al. (2009) demonstrated 47.6% to be eligible for PKR based on the wear pattern of the knee. Combining these numbers, it seems justified to perform PKR in hospitals with knee arthroplasty populations of 50–100 patients or larger, taking into account that each surgeon should perform at least 10–13 PKRs per year.

In conclusion, data from the Dutch Arthroplasty Register confirm that the absolute hospital PKR volume should be high (in this study > 58 PKRs annually) to achieve the lowest risk of revision. Proportional hospital PKR volume did not show an effect on implant survival, indicating that incorporation of PKR in different hospital practices is possible as long as the absolute hospital PKR volume is high enough. The latter can be easily achieved since more than 40% of the arthroplasty population actually seems to be eligible for PKR. In addition, by using in-hospital referral the desired absolute and proportional surgeon levels can also be easily achieved.
